# A new focus of *Aedes japonicus japonicus* (Theobald, 1901) (Diptera, Culicidae) distribution in Western Germany: rapid spread or a further introduction event?

**DOI:** 10.1186/1756-3305-5-284

**Published:** 2012-12-07

**Authors:** Helge Kampen, Dorothee Zielke, Doreen Werner

**Affiliations:** 1 Friedrich-Loeffler-Institut, Federal Research Institute for Animal Health, Suedufer 10, Greifswald, Insel Riems, 17493, Germany; 2 Leibniz-Centre for Agricultural Landscape Research, Eberswalder Str. 84, Muencheberg, 15374, Germany

**Keywords:** *Aedes japonicus japonicus*, Cemeteries, Distribution focus, Spread, Western Germany

## Abstract

**Background:**

The Asian bush mosquito, *Aedes japonicus japonicus*, a potential vector of several viruses, was first detected in Germany in 2008 on the Swiss-German border. In the following years, this invasive species apparently succeeded in establishing populations in southern Germany and in spreading northwards. In 2011, its distribution area already covered large areas of the federal state of Baden-Wurttemberg, and its northernmost German collection point was reported to be close to Stuttgart. Several independent submissions to our laboratories of *Ae*. *j*. *japonicus* specimens in July 2012, originating from the same area in the federal state of North Rhine-Westphalia, western Germany, prompted us to carry out an immediate surveillance in this region in the expectation of finding a further distribution focus of *Ae*. *j*. *japonicus* in Germany.

**Methods:**

After inspecting the places of residence of the collectors of the submitted mosquito specimens, all kinds of water containers in 123 cemeteries in surrounding towns and villages were checked for mosquito developmental stages. These were collected and kept to produce adults for morphological species identification. One specimen per collection site was identified genetically by COI sequence analysis.

**Results:**

*Aedes j*. *japonicus* adults and immature stages were found in 36 towns/villages that were checked (29%) over an area of approximately 2,000 km^2^ in southern North Rhine-Westphalia and northern Rhineland Palatinate. The species could not be demonstrated further south when monitoring towards the northernmost previous collection sites in southern Germany. It therefore remains to be elucidated whether the species has entered western Germany from the south, from Belgium in the west where it has been demonstrated to occur locally since 2002, or through a new introduction.

**Conclusions:**

*Aedes j*. *japonicus* is obviously much more widely distributed in Germany than previously thought. It appears to be well adapted, to have a strong expansion tendency and to replace indigenous mosquito species. Thus, a further spread is anticipated and elimination seems hardly possible anymore. The vector potency of the species should be reason enough to thoroughly monitor its future development in Germany.

## Background

Next to the Asian tiger mosquito *Aedes albopictus*, the Asian bush mosquito, *Aedes japonicus japonicus* (Diptera, Culicidae), is one of the most expansive invasive mosquito species in the world [1]. After the brief interception of *Ae*. *j*. *japonicus*, together with *Ae*. *albopictus*, in New Zealand in the early 1990s [2], the species was first reported to have established outside its native distribution area in 1998 in the eastern US [3]. It was probably introduced by the international used tyre trade some years before that, and several times independently [4,5]. *Aedes j*. *japonicus* has spread considerably since then and is now widely distributed in the eastern part of North America, including Canada [6]. In central Europe, larvae were found in 2000 on the premises of a used tyre trading company in France, but these were quickly eliminated [7]. In 2002, specimens were also collected at two sites in a town in central Belgium, again associated with tyre trading companies. The species still colonized that area in 2003 and 2004 and was present in 2007 and 2008 during a national Belgian mosquito survey [8]. As specimens were never collected elsewhere in Belgium during this survey, the species was considered to be confined to the surroundings of the two companies and control did not take place until 2012. In 2008, *Ae*. *j*. *japonicus* appeared in northern Switzerland, and immature stages were also discovered at several sites on German territory along the Swiss-German border [9]. Monitoring carried out in the German federal state of Baden-Wurttemberg in 2009 and 2010 showed the mosquito to occur in a broader region of southern Germany along the Swiss border [10]. Another small study restricted to a limited area south of Stuttgart demonstrated *Ae*. *j*. *japonicus* in mid-2010 some 80 km north of the South German distribution area [11]. Three hitherto unpublished findings from 2011 of five females outside the described distribution areas were made in the cities of Stuttgart, Freiburg and Breisach, approx. 20 km west of Freiburg (own results).

*Aedes j*. *japonicus* is not only an aggressive biter but a suspected or known vector of various viruses. It has repeatedly been found infected in the field with West Nile virus in the US [12] and efficiently transmits this virus in the laboratory [13,14]; it was shown to be able to transmit Japanese encephalitis virus horizontally and vertically [15] and to be vector-competent for LaCrosse [16], eastern equine encephalitis [17] and St. Louis encephalitis viruses [18] under experimental conditions. Only recently it has also been demonstrated to be vector-competent for chikungunya and dengue viruses in the laboratory, two disease agents that have emerged and re-emerged in southern Europe [19].

In July 2012, seven specimens of *Ae*. *j*. *japonicus* were submitted to our laboratories for species identification. In four cases, they had been collected indoors while attacking humans. Three of the mosquitoes were sent by one and the same person, the other four by different persons independently, but all of them from the larger Bonn area in the federal state of North Rhine-Westphalia, West Germany. Taking these coincidences to be a strong hint that there could be a local *Ae*. *j*. *japonicus* population, a small monitoring programme was promptly initiated including an inspection of the mosquito collection sites and the screening of cemeteries in that area for immature stages.

## Methods

The field work of the study was carried out in August 2012 within less than two weeks. To start with, the immediate surroundings of all five sites where the submitted *Ae*. *j*. *japonicus* adults had been collected were inspected for mosquito breeding sites. Water containers of every kind, such as flower-pot saucers, rain water barrels and paddling pools, were checked in the collectors’ gardens as well as in the neighbourhood and in the nearest cemeteries, similar to the procedure described by Schaffner *et al*. [9]. To check for further distribution areas, flower vases, watering cans, stone basins and other potential breeding sites were examined in the cemeteries of towns and villages in all geographic directions from the initial detection sites. Once the bush mosquito was found in an inspected cemetery, further villages were checked in distances between 5 and 20 km, following the direction of larval presence. All visited sites were geo-referenced.

Mosquito larvae and pupae were collected by sieves and pipettes and transferred to labelled glas jars with screw tops filled with water from their breeding sites, where they were kept until adult emergence. For the purpose of collecting the emerged adults, a jar was put into a mesh insect cage where its top was removed. The mosquitoes were then collected from the cage by an aspirator and killed by deep freezing (−20°C) for 15 to 20 min. They were identified morphologically using the determination keys by Tanaka *et al*. [20], Schaffner *et al*. [21] and Becker *et al*. [22]. For genetic species confirmation, the cytochrome c oxidase subunit I (COI) gene of one specimen per collection site was PCR-amplified by primers PanCuli-COX1-211 F (5’-ATCATAATTGGTGGGTTTGGWAAYTGA-3’) and PanCuli-COX1-727R (5’-TATAAACTTCDGGRTGNCCAAARAATC-3’) and sequenced according to standard protocols [23].

## Results and discussion

Mosquito larvae were found on all premises inspected. *Aedes j*. *japonicus* could be detected in flower vases, flower-pot saucers, watering cans, rain water barrels and a baby paddling pool, either as immature stages or as freshly emerged adults, in the gardens of four of the five senders of the original mosquitoes or in the immediate surroundings of their homes. Only at the place of residence of the fifth sender, corresponding to the easternmost collection site, no bush mosquitoes were discovered during a site inspection.

Whilst *Ae*. *j*. *japonicus* adults can be easily identified in the field, the larvae can also be differentiated from those of indigenous species with some reliability. They are brownish-yellow or darker, have a quite slender appearance and a moderately long siphon. Even in an advanced larval stage, their head capsule has no bright areas on the dorsal side giving the impression of a dark, more or less round shield. Eyes and mouthparts are not distinctly visible with the naked eye. The larvae spend long periods of time on the ground of their breeding site with little movement, often between debris if present, but if they move they wiggle more intensely and worm-like than the larvae of most indigenous mosquito species ([20,24], own observation).

Using these identification criteria, the distribution area of *Ae*. *j*. *japonicus* was provisionally defined whilst in the field, by checking vases and other water containers in cemeteries, which had proved to be a suitable approach in other studies [9,25]. Only after the collecting trips, which took several days each, the emerged mosquitoes were properly identified to species in the laboratory using a dissecting microscope. The GenBank accession numbers for the *Ae*. *j*. *japonicus* COI sequences generated for confirmation of the morphological determination are JX888952-94.

**Figure 1:**
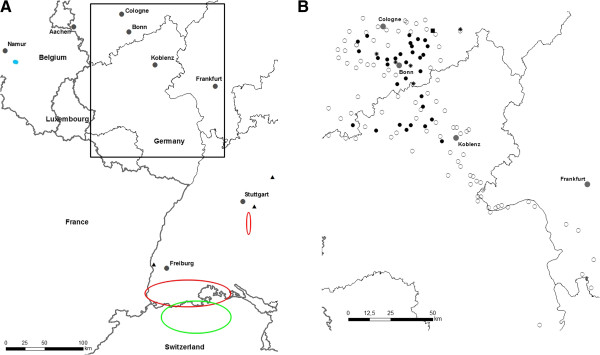
**Distribution areas of *Ae. j. japonicus *in central Europe. a**: Overview map: area encircled red: distribution areas in South Germany according to Becker *et al*. [10] and Schneider [11]; area encircled green: distribution area in northern Switzerland according to Schaffner *et al*. [9]; blue dots: local occurrence in Belgium according to Versteirt *et al*. [8]. ▲: Own unpublished accidental findings of *Ae*. *j*. *japonicus* outside the previously described German distribution area. Box: see Figure 1b. **b**: Detailed map with collection sites of this study. ✱: Places of residence of *Ae*. *j*. *japonicus* senders, ●: cemeteries positive for *Ae*. *j*. *japonicus*, ○: cemeteries negative for *Ae*. *j*. *japonicus*, ■: rain water barrel positive for *Ae*. *j*. *japonicus*

In total, out of 123 cemeteries inspected, 35 were found to be infested by *Ae*. *j*. *japonicus*. In some towns/villages where cemeteries were positive, larvae of the bush mosquito were also found in water containers away from the cemeteries. In one village without a cemetery, larvae were detected in a rain water barrel. Thus, *Ae*. *j*. *japonicus* could be demonstrated in 36 of 124 towns/villages checked, accounting for 29%. The area where *Ae*. *j*. *japonicus* occurred in western Germany covered approximately 2,000 km^2^ (Figure 1).

Considering the area of distribution, it can be assumed that the introduction of *Ae*. *j*. *japonicus* into western Germany dates back to earlier years although complaints only started in 2012 and immature stages could not be found in the infested area during occasional inspections of cemeteries in 2011 and in May 2012.

The origin of the infestation could not be resolved. Of the most obvious possibilities, a northward spread from infested areas in southern Germany (shortest direct line distance between the newly discovered distribution area and the northernmost point of previously known occurrence: approx. 250 km) or an eastward spread from the infestation area in Belgium (shortest direct line distance between the newly discovered distribution area and the Belgian town with the two infested tyre trading companies: approx. 150 km), neither could be verified under the hypothesis that a continuous, more or less linear distribution had taken place. Regions with no documentation of *Ae*. *j*. *japonicus* appear to exist between all three distribution areas, although distribution zones with low population densities might have been missed. Prevailing modes of dispersal, active or passive, and routes, e.g. along valleys or rivers, are unknown for this mosquito species. An accidental finding of *Ae*. *j*. *japonicus* larvae in 2011, however, close to the town of Schwaebisch-Hall in northeastern Baden-Wurttemberg rather suggests an active invasion from the south.

A third possibility is an additional importation event of the bush mosquito into central Europe, with subsequent establishment. The used tyre trade is no big business in Germany, but it does exist on a limited scale. Efforts to identify tyre trading companies in the newly recognized infested area are under way as well as molecular (microsatellite DNA) analyses of the mosquitoes collected in western Germany to elucidate their genetic relationships to populations from other European and non-European distribution areas.

Although the mosquito collection data from western Germany are not appropriate for ecological and epidemiological analyses, the strong impression arose that *Ae*. *j*. *japonicus* tends to displace and replace indigenous mosquito species, just as it appears to do in the US [26]. Except for the peripheral sites of the infested area, where sometimes only one or two water containers with *Ae*. *j*. *japonicus* larvae were discovered in a cemetery, often only very few water containers, or no container at all, were occupied by larvae of other mosquito species when *Ae*. *j*. *japonicus* immature stages were present. This subject needs further investigation.

A more detailed and systematic monitoring for *Ae*. *j*. *japonicus* in western Germany, addressing questions including indigenous mosquito species displacement and speed of spread, will be carried out in 2013, taking into account the strategies suggested by the ECDC in their guidelines for the surveillance of invasive mosquitoes in Europe [27].

## Conclusion

*Aedes j*. *japonicus* is not considered an important vector of disease agents under natural conditions in its native geographic distribution area, Japan, Korea, Taiwan and southern China, but has been shown to be vector-competent for several highly pathogenic viruses in the laboratory. Thorough observation of the species in its new distribution areas in Europe is therefore recommended, the more so as it feeds on both birds and humans and thus might serve as a bridge vector for zoonotic disease agents reservoired by birds [28].

Taking into account the considerable distribution areas of *Ae*. *j*. *japonicus* in Switzerland and Germany, it is hardly conceivable that this invasive mosquito species could again be eliminated from central Europe. On the contrary, it seems to be very well adapted to European conditions, and a further spread is likely.

## Abbreviations

COI: Cytochrome c oxidase subunit I.

## Competing interests

The authors declare that they have no competing interests.

## Authors’ contributions

HK designed the study, collected the mosquitoes, carried out the molecular mosquito identification and drafted the manuscript. As part of her doctoral thesis, DZ collected the mosquitoes, performed the morphological identifications and was involved in writing the manuscript. DW designed the study, collected the mosquitoes, conducted a significant part of the morphological mosquito identification and contributed to the drafting of the manuscript. All authors read and approved the final version of the manuscript.
